# Scoring Selection Criteria Including Total Tumour Volume and Pretransplant Percentage of Lymphocytes to Predict Recurrence of Hepatocellular Carcinoma after Liver Transplantation

**DOI:** 10.1371/journal.pone.0072235

**Published:** 2013-08-21

**Authors:** Chuan Li, Tian-Fu Wen, Lu-Nan Yan, Bo Li, Jia-Ying Yang, Ming-Qing Xu, Wen-Tao Wang, Yong-Gang Wei

**Affiliations:** Division of Liver Transplantation, West China Hospital of Sichuan University, Chengdu, Sichuan Province, China; The University of Hong Kong, China

## Abstract

**Aim:**

The selection criteria for patients with hepatocellular carcinoma (HCC) to undergo liver transplantation should accurately predict posttransplant recurrence while not denying potential beneficiaries. In the present study, we attempted to identify risk factors associated with posttransplant recurrence and to expand the selection criteria.

**Patients and Methods:**

Adult patients with HCC who underwent liver transplantation between November 2004 and September 2012 at our centre were recruited into the current study (N = 241). Clinical and pathological data were retrospectively reviewed. Patients who died during the perioperative period or died of non-recurrence causes were excluded from this study (N = 25). All potential risk factors were analysed using uni- and multi-variate analyses.

**Results:**

Sixty-one recipients of 216 qualified patients suffered from recurrence. Similar recurrence-free and long-term survival rates were observed between living donor liver transplant recipients (N = 60) and deceased donor liver transplant recipients (N = 156). Total tumour volume (TTV) and preoperative percentage of lymphocytes (L%) were two independent risk factors in the multivariate analysis. We propose a prognostic score model based on these two risk factors. Patients within our criteria achieved a similar recurrence-free survival to patients within the Milan criteria. Seventy-one patients who were beyond the Milan criteria but within our criteria also had comparable survival to patients within the Milan criteria.

**Conclusions:**

TTV and L% are two risk factors that contribute to posttransplant recurrence. Selection criteria based on these two factors, which are proposed by our study, expanded the Milan criteria without increasing the risk of posttransplant recurrence.

## Introduction

Hepatocellular carcinoma (HCC) is the sixth most common neoplasm and the third leading cause of cancer-related death worldwide [1]. Due to the high prevalence of hepatitis B infection, China alone accounts for approximately 55% of HCC cases worldwide [2]. Liver transplantation is one of the curative treatments for patients with HCC. However, HCC recurrence after liver transplantation is a complication that negatively impacts the long-term survival of recipients. In 1996, Mazzaferro and colleagues [3] proposed the Milan criteria (single tumour with a maximum diameter ≤5 cm; or up to three tumours with none more than 3 cm), which are widely accepted as the gold standard for selecting the best HCC candidates for liver transplantation. Patients with HCC within the Milan criteria and who undergo liver transplantation can achieve a similar long-term survival to patients with benign liver disease. However, the Milan criteria are too stringent and may deny HCC patients who may benefit from liver transplantation. Subsequently, Yao et al [4] confirmed appropriate expansion of the Milan criteria did not negatively impact HCC patient survival and expanded the Milan criteria to the University of California, San Francisco (UCSF) criteria (single tumour up to 6.5 cm in maximum diameter; or up to three tumours with none larger than 4.5 cm and with a total tumour diameter no more than 8 cm). However, the UCSF criteria are still too stringent. How to expand the selection criteria without increasing the risk of posttransplant recurrence is still a highly discussed topic in the transplant field. In the present study, we attempted to identify the risk factors associated with recurrence after liver transplantation.

## Patients and Methods

### Study Group

Patients with HCC who underwent liver transplantation between November 2004 and September 2012 were recruited into the present study (N = 241). The current selection criteria for liver transplantation are up to 9 cm in total tumour diameter without macrovascular invasion or exhepatic metastasis, regardless of the tumour number. The patients who died during the perioperative period and who died of non-tumour related causes were excluded from the study (N = 25). According to the patient outcomes, the patients were divided into a recurrence group and non-recurrence group. At the time of surgery, written consent was obtained from all patients for their information to be stored in the hospital database and used for research. All of the liver transplantations and the present study were approved by the ethics committee of West China Hospital, Sichuan University. The ethics committee also approved the retrospective analysis of existing patient data without additional informed consent for the current study because we received written consent for scientific research from all patients at the time of surgery and there was a low risk of breaching confidentiality.

### Donor Selection

Living donors were required to be ABO blood type-compatible close relatives. Serological testing for hepatitis virus and human immunodeficiency virus antibodies as well as testing for other acute or chronic diseases were required to be negative. Volumetric computed tomography with contrast was utilised to evaluate the right lobe of the donor’s liver. The right hepatic lobe without the middle hepatic vein was required to be at least 0.8% of the recipient’s weight, and at least 40% of the right hepatic lobe was required to remain for the donor. Magnetic resonance cholangiopancreatography was performed to assess the anatomy of the biliary tree.

### Immunosuppression and Antivirus Protocols

Postoperative immunosuppressive maintenance consists of Calcineurin Inhibitor (tacrolimus or cyclosporine A), mycophenolate mofetil and steroids. Steroids were decreased as early as possible to reduce the risk of HCC recurrence. Steroid pulse therapy was administered to patients with rejection. For patients with positive hepatitis B surface antigen, the antivirus therapy includes Lamivudine and hepatitis B immune globin.

### Follow up and Definitions

After liver transplantation, recipients were regularly monitored via serum alpha fetoprotein (AFP), visceral ultrasonography or computed tomography or magnetic resonance imaging and chest radiography every 3 months. Bone scintigraphy was performed whenever HCC recurrence was suspected. Postoperative recurrence was defined as positive imaging findings compared to preoperative examinations and newly rising tumour markers. All recipients were regularly followed up to recurrence, death or termination of the study.

Model for end-stage liver disease score was calculated using the following formula: MELD score = 9.57×Ln creatinine (mg/dL)+11.2×(Ln INR)+3.78×Ln bilirubin (mg/dL)+6.43 [5]. Total tumour volume (TTV) was calculated as the sum of the volumes of all tumours (4/3 πr^3^, where r is the maximum radius of each tumour) [6].

### Statistical Analysis

All analyses were performed using SPSS 16.0 for windows. Continuous variables were presented as the mean ± SD and compared using one-way analysis of variance. Categorical variables were analysed using the Chi-squared test or Fisher’s exact test. The Kaplan-Meier method with log-rank test was performed to compare the recurrence-free survival of different groups. A *P* value less than 0.05 was considered to be statistically significant. The diagnostic accuracy of the identified risk factors was evaluated using receiver operating curves (ROC). To assess the ability of different models to predict HCC recurrence after liver transplantation, our analysis was performed using the c-statistic equivalent to the area under the ROC curve (AUC).

## Results

**Figure 1:**
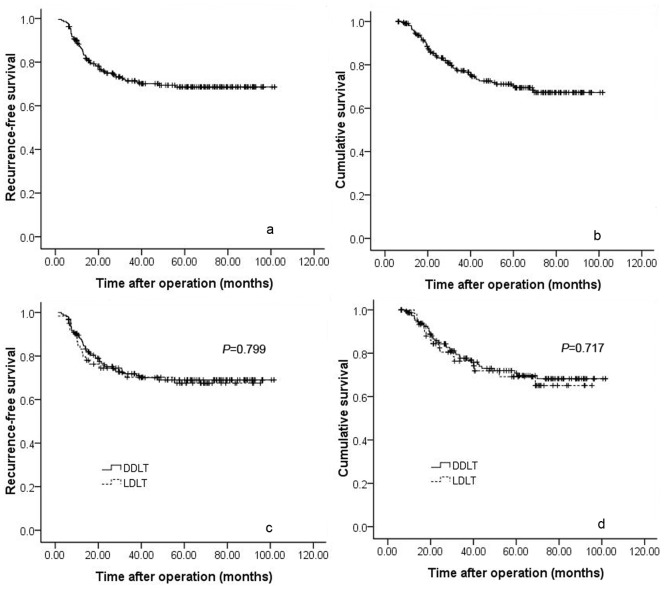
The recurrence-free survival (figure 1a) and long-term survival (figure 1b) curves for all of the patients. Recurrence-free survival (figure 1c, *P* = 0.799) and overall long-term survival curves (figure 1d, *P* = 0.717) for patients undergoing LDLT vs. DDLT.

A total of 241 recipients were included in the present study. Twenty-five recipients, including 23 patients who died during the perioperative period, 1 patient who died of suicide and 1 patient who died of infection, were excluded from the current study. The remaining 216 patients included 60 patients who underwent living donor liver transplantation (LDLT) and 156 patients who underwent deceased donor liver transplantation (DDLT). The mean follow-up period was 47.87±28.07 months. The mean recipient age was 47.22±9.29 years, whereas the mean donor age was 33.90±8.74 years. The mean TTV was 115.91±108.75 cm^3^. The mean MELD score was 11.72±5.78. In the present study, 117 patients were within the UCSF criteria, whereas 93 patients were within the Milan criteria. During the follow-up period, 61 patients suffered from HCC recurrence, including 18 LDLT recipients and 43 DDLT recipients. Of the 61 patients, 56 patients, including 17 LDLT patients and 39 DDLT patients, died of tumour recurrence. The overall 1-, 3- and 5-year recurrence-free survival rates were 86.2%, 70.8% and 68.6% (figure 1a), respectively, whereas the 1-, 3- and 5-year long-term survival rates were 97.6%, 77.3% and 70.3% (figure 1b), respectively.

### Comparison of LDLT versus DDLT for Patients with HCC

We compared the outcomes of patients who underwent LDLT versus DDLT. As shown in table 1, the LDLT group had more female donors than the DDLT group. Except for the difference in donor gender, the LDLT and DDLT groups were comparable with respect to age, tumour size, tumour differentiation, and other metrics. The 1-, 3- and 5-year survival rates of LDLT recipients were 81.4%, 70.2% and 67.5% versus 87.5%, 71.9% and 69% for DDLT recipients, respectively. No significant differences were observed (figure 1c, *P* = 0.799). The overall long-term survival rates for the two groups were also similar (98.3%, 76.3% and 69.2% for LDLT patient and 97.3%, 77.7% and 70.8% for DDLT patients, respectively; figure 1d, *P* = 0.717).

**Table 1:** Comparison of the demographic data for the LDLT and DDLT groups.

Variables	DDLT (N = 156)	LDLT (N = 60)	*P*
**Donor variables**			
Age (years)	33.77±7.49	34.23±11.43	0.727
Gender (female)	6	20	<0.001
BMI (kg/m^2^)	22.73±2.19	22.63±1.89	0.749
**Recipient variables**			
Age (years)	47.99±9.60	45.23±8.18	0.050
Gender (female)	18	6	0.747
BMI (kg/m^2^)	22.84±2.88	23.32±3.61	0.314
AFP (ng/ml)	512.54±513.96	536.61±505.62	0.757
Tumour size (cm)	5.18±2.25	5.34±2.23	0.632
TTV (cm^3^)	114.54±109.93	119.47±106.45	0.767
Multiple tumours	28	6	0.151
Microvascular invasion	45	25	0.071
Differentiation (poor/moderate/well)	25/106/25	7/41/12	0.620
MELD	11.92±6.17	11.22±4.60	0.426

### Factors Associated with Posttransplant Recurrence

**Figure 2:**
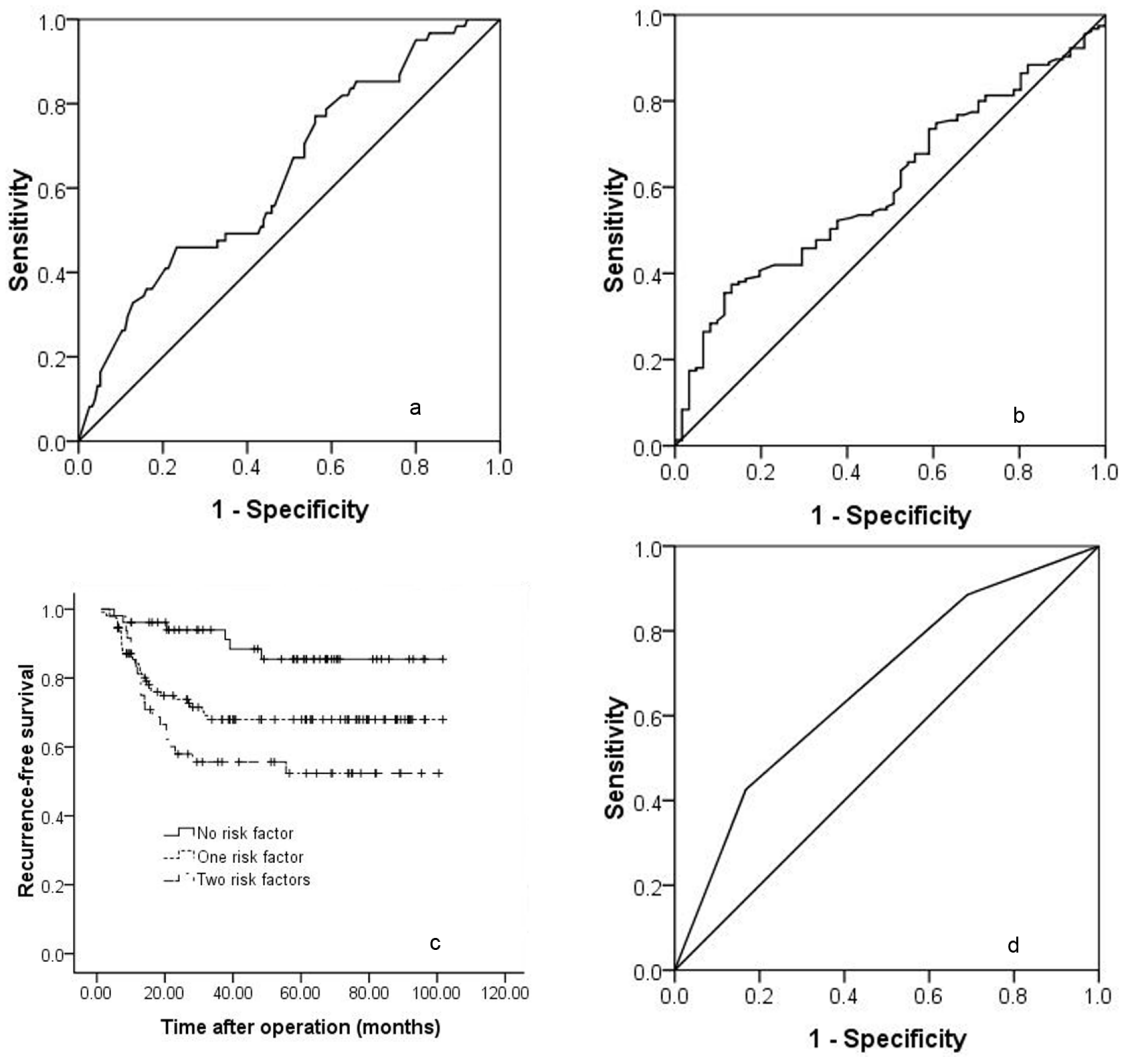
Receiver operating curves for TTV (figure 2a) and L% (figure 2b). Cumulative survival curves for patients with 0, 1 or 2 of the risk factors that were confirmed by multivariate analysis (figure 2c, *P*<0.001). Receiver operating curve for the predictive risk factors that were confirmed by the multivariate analysis (figure 2d).

As listed in table 2, the preoperative percentage of lymphocytes (L%), total tumour size, TTV, microvascular invasion and measurements other than those in the UCSF criteria were potential risk factors in the univariate analyses, whereas multiple tumours, percentage of neutrophils (N%), neutrophil-to-lymphocyte ratio (NLR) and tumour differentiation did not show prognostic power. However, as shown in table 3, only TTV and L% were independent risk factors in the multivariate analysis using the Cox regression model. The ROC curve analysis showed the best cut-off values for TTV and L% were 172 cm^3^ and 30.2%, respectively (figure 2a and figure 2b). In the present study, we chose 30% as the cut-off value for L%.

**Table 2:** Univariate analyses for the risk factors of postoperative recurrence.

Variables	Non-recurrence group	Recurrence group	*P*
**Donor variables**			
Age (years)	33.85±8.56	34.02±9.25	0.901
Gender (female)	20	6	0.533
BMI (kg/m2)	22.82±2.14	22.41±2.02	0.203
**Recipient variables**			
Age (years)	47.67±9.44	46.10±8.88	0.214
Gender (female)	20	4	0.214
BMI (kg/m2)	22.91±3.18	23.13±2.92	0.652
AFP (ng/ml)	496.51±514.31	576.96±500.56	0.298
N%	64.77%±13.40%	61.84%±14.36%	0.157
L%	27.94%±14.00%	22.83%±10.37%	0.010
N/L	4.12±5.16	4.10±2.73	0.974
Tumour size (cm)	4.95±2.24	5.93±2.10	0.003
TTV	102.61±101.06	149.71±120.60	0.004
Multiple tumours	25	9	0.803
Microvascular invasion	45	25	0.091
Differentiation (poor/moderate/well)	29/105/21	8/42/11	0.496
MELD	11.98±6.08	11.07±4.91	0.296
LDLT	42	18	0.722
Beyond the Milan criteria	72	21	0.108
Beyond the UCSF criteria	90	27	0.067

**Table 3:** Multivariate analysis for the risk factors of postoperative recurrence.

Variables	OR	95% CI	*P*
TTV	1.002	1.000–1.005	0.028
L%	0.975	0.954–0.998	0.032

### A Preoperative Prognostic Score Model

**Figure 3:**
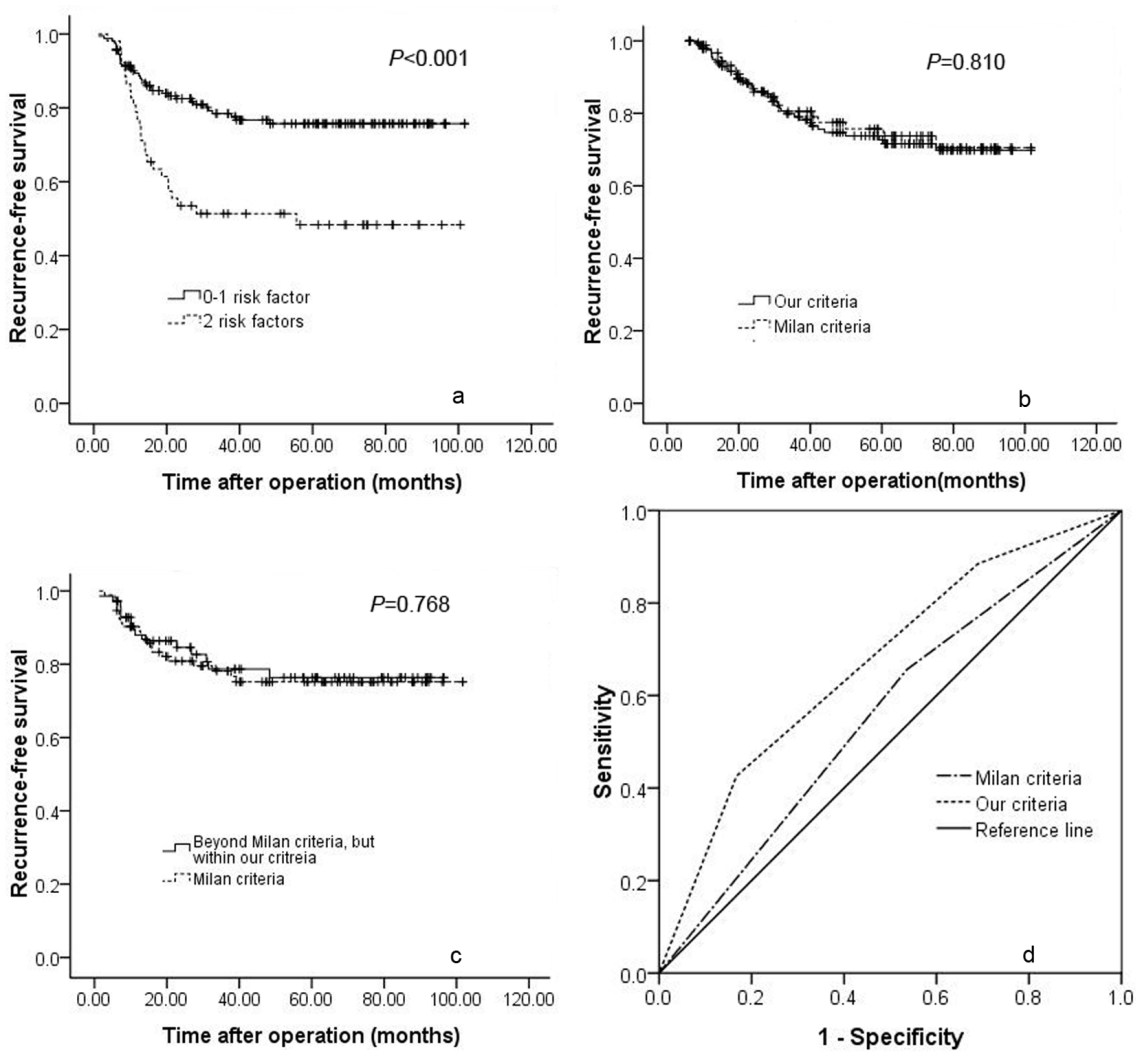
Cumulative survival curves of patients with 0–1 and 2 of the risk factors that were confirmed by multivariate analysis (figure 3a, *P*<0.001). Recurrence-free survival curve of patients within our criteria vs. the Milan criteria (figure 3b, *P* = 0.810). Recurrence-free survival curve for patients beyond the Milan criteria but within our criteria vs. patients within the Milan criteria (figure 3c, *P* = 0.768). Comparison of the AUCs of our criteria and the Milan criteria (figure 3d).

A simple preoperative prognostic score was derived from the results of the Cox regression analysis. Each above-mentioned risk factor was given a score of 1, and patients were stratified into 3 categories according to the number of risk factors. As presented in table 4, if patients did not have any risk factors, the prognostic score was 0. If patients had any one of the above-mentioned risk factors, the prognostic score was 1. If patients had both of the two risk factors, the prognostic score was 2. In the present study, 55 patients did not have any risk factor, 109 patients had one risk factor and 52 patients had 2 risk factors. As shown in figure 2c, the 1-, 3- and 5-year recurrence-free survival rates of patients with two, one and no risk factors were 76.9%, 51.4% and 48.3%; 85.6%, 71.3% and 71.3%; and 94.5%, 89.8% and 84.3%, respectively. A significant difference was observed (*P*<0.001). When the number of risk factors was analysed and confirmed by multivariate analysis with a ROC, the best cut-off value for the number of risk factors was determined to be greater than 1 (figure 2d). Because we excluded patients with macrovascular invasion and/or exhepatic metastasis from this study and the maximum tumour size was 9 cm (the TTV was 382 cm^3^) in this study, we proposed a new prognostic score model to predict HCC recurrence after liver transplantation as follows: 1) the TTV of HCC patients is no more than 172 cm^3^, regardless of the preoperative percentage of lymphocytes; 2) if the TTV of HCC patients is greater than 172 cm^3^ (about one tumour up to 7 cm in maximum diameter) and less than 382 cm^3^, the preoperative percentage of lymphocytes should be no less than 30%; 3) there is no macrovascular invasion or exhepatic metastasis. A total of 164 patients fulfilled our criteria, including 93 patients who were within the Milan criteria and 71 patients who were beyond the Milan criteria. The 1-, 3- and 5-year recurrence-free survival rate of patients within our criteria was much better than those beyond our criteria (figure 3a, 88.7%, 78.4% and 75.7% versus 76.9%, 51.4% and 48.3%, respectively; *P*<0.001). We also compared the recurrence-free survival rates of patients within our criteria with patients within the Milan criteria. As shown in figure 3b, the 1-, 3- and 5-year recurrence-free survival rates of patients within our criteria were similar to those of patients within the Milan criteria (88.7%, 78.4% and 75.7% versus 89.1%, 78.1% and 75.2%, respectively; *P* = 0.810). Seventy-one patients beyond the Milan criteria but within our criteria benefited from liver transplantation. The 1-, 3- and 5-year recurrence-free survival rates of patients beyond the Milan criteria but within our criteria were 88%, 78.7% and 76.3%, respectively, which were similar to patients within the Milan criteria (figure 3c, *P* = 0.768).We also compared the predictive ability of our criteria and the Milan criteria by assessing the AUC of the two criteria. As shown in figure 3d, the AUC of our criteria was significantly higher than that of the Milan criteria (0.670 vs. 0.560, *P* = 0.002).

**Table 4:** Independent predictors and assigned risk score points.

Variables	Risk score points
TTV≤172 cm^3^	0
TTV>172 cm^3^	1
L%≥30%	0
L%<30%	1

## Discussion

Liver transplantation is perceived as one of the curative treatments for patients with HCC. Because deceased donor grafts are a public resource, how to efficiently and fairly allocate this resource is an issue that deserves further study. Although a number of investigations have confirmed HCC patients within the Milan criteria who undergo liver transplantation have excellent outcomes, these strict selection criteria may deny other HCC patients who may benefit from liver transplantation but do not meet the Milan criteria. [7], [8], [9] Recently, even Mazzaferro and colleagues [10] proposed up-to-seven criteria to expand the Milan criteria. In the present study, we confirmed TTV and preoperative percentage of lymphocytes are two independent risk factors for HCC recurrence in patients undergoing liver transplantation and proposed a prognostic scoring model based on these two risk factors. According to our model, 32.87% of patients who were beyond the Milan criteria still achieved a similar outcome to those within the Milan criteria.

A number of selection criteria of patients with HCC for liver transplantation used tumour size and tumour number as two variables to select patients. [3], [4] However, our study suggests the TTV of HCC patients was a useful prognostic predictor for patients with HCC undergoing liver transplantation. Although TTV is also mainly dependent on the maximum tumour size, the tumour number did not play a key role in selecting patients. For instance, both the Milan and UCSF criteria suggest the tumour number should not be greater than 3. Accordingly, patients with more than 3 small nodules were denied by both the Milan and UCSF criteria. However, these patients may be included in our model. In our practice, a patient with 6 small tumours received liver transplantation and survived without recurrence. Therefore, our model may be beneficial to patients with multiple small tumours. Furthermore, TTV did not define a maximum tumour size for each tumour. The TTV only emphasised the total volume of HCC. For example, patients with two HCC tumours, of which one is 5 cm, are also denied transplantation by both the Milan and UCSF criteria. However, these patients may also have a chance to undergo liver transplantation according to our criteria. Similar to our study, Toso et al [6] also confirmed TTV is an independent risk factor for patients with HCC following liver transplantation. They suggested the best cut-off value for TTV was 115 cm^3^. [6] In their study, they measured the tumour size using pathological data. [6] However, our results were based on imaging findings. Tumours may shrink without blood supply after resection, which may explain why Toso et al’s cut-off value is smaller than our criteria. In the present study, pathological data were unavailable before the surgery. Accordingly, our model may be a better reflection of actual clinical practice. Additionally, a TTV of 172 cm^3^ is approximately 7 cm in diameter for a single tumour. If the preoperative percentage of lymphocytes is not less than 30%, the TTV could be expanded to 382 cm^3^ (about 9 cm in diameter for a single tumour). Therefore, our model is not only an expansion of the Milan criteria but also an expansion of the UCSF criteria.

Different from previous investigations, the NLR did not show prognostic power in the present study, whereas the preoperative percentage of lymphocytes was associated with recurrence in the multivariate analysis. Halazun et al [11] confirmed HCC patients with a high NLR undergoing liver transplantation had worse recurrence-free and long-term survival rates than those with a low preoperative NLR. Subsequently, a number of investigations suggested preoperative NLR may be a useful predictor for patients with HCC undergoing liver resection and radio frequency. [12], [13] Halazun et al [11] hypothesised the potential explanation for NLR in predicting HCC patient outcomes as follows: First, patients with a high preoperative NLR may have a high percentage of neutrophils and a low percentage of lymphocytes; however, previous investigations suggest neutrophils could secret vascular endothelial growth factor (VEGF) which could lead to angiogenesis and improve tumour growth [14], [15]; Second, after liver transplantation, the antitumour immune response largely depends on the lymphocyte system. [16], [17] A low preoperative percentage of lymphocytes may weaken the antitumour response of patients with a high NLR. However, recently, Motomura and colleagues [18] confirmed the VEGF, interleukin-8 (IL-8), IL-17, CD68 and CD163 levels were similar between patients with low and high preoperative NLRs who underwent liver transplantation. Patients with a high NLR have a high level of IL-17 in the serum and peritumoural tissues. [18] CD163-positive tumour-associated macrophages correlated significantly with IL-17-producing cells. [18] According to Motomura et al’s [18] results, the NLR’s ability to predicts tumour recurrence may not be through angiogenesis but via the inflammatory tumour microenvironment. Our study also confirmed the preoperative percentage of neutrophils was not different between the recurrence and non-recurrence groups. Western et al [19] also suggested preoperative NLR cannot predict the outcomes of patients with HCC undergoing liver resection. We hypothesise the correlation between preoperative percentage of lymphocytes and postoperative recurrence may be due to the weakened antitumour response in patients with a low percentage of lymphocytes. Eto et al [20] even suggested posttransplant administration of donor lymphocyte infusions may induce potential antitumour immunity to solid tumours for patients with haematological malignancy. However, the detailed mechanism of correlation between percentage of lymphocytes and postoperative recurrence is unclear and requires further study.

Performing LDLT for patients with HCC remains controversial. LDLT is referred to as “fast track” transplantation because of the shorter waiting time compared to DDLT. Due to the short waiting time, more patients with aggressive tumours receive LDLT, whereas these patients may drop out from the waiting list due to tumour regression when waiting for a deceased donor liver graft. [21] Moreover, LDLT needs to preserve the native inferior vena cava as well as the greater length of the bile duct and hepatic artery. Accordingly, some investigators suggest LDLT could not radically remove all tumour cells. [22], [23] LDLT transplants a partial liver graft, which is smaller than the graft used in DDLT. Animal and clinical studies have demonstrated postoperative excessive portal venous flow and transient portal hypertension may lead to graft injury, which accompanies angiogenesis and cell migration. [24], [25] Additionally, rapid liver regeneration during the early period after LDLT is also an angiogenesis-associated phenomenon. [26] As a complex surgical procedure, more manipulation during LDLT may also lead to tumour metastasis to exhepatic sites via the hepatic vein. For the above-mentioned reasons, LDLT for patients with HCC may have poorer outcomes compared to DDLT. However, recently, a meta-analysis performed by Liang et al [27] indicates the outcomes of patients with HCC undergoing LDLT were similar to those of patients who received DDLT, especially for those within the Milan criteria. Our study further confirmed LDLT can achieve similar outcomes to DDLT for patients with HCC when using expanded selection criteria. However, interestingly, another meta-analysis performed by Grant et al [28] suggested the recurrence-free survival rate of patients who underwent LDLT was poorer than that of patients who underwent DDLT. Previous investigations suggested macrovascular invasion and tumour size were the predominant factors that contributed to postoperative recurrence rather than the differences in the liver grafts. Our study also confirmed preoperative percentage of lymphocytes and TTV were independent risk factors for posttransplant recurrence.

Some investigations suggested microvascular invasion predict recurrence after liver transplantation [29], [30]. However, in the present study, microvascular invasion did not show any prognostic power. Using microvascular invasion to predict posttransplant recurrence remains controversial. Shah et al [31] confirmed macrovascular invasion but not microvascular invasion adversely impacts outcomes for liver transplant recipients. Ataide et al [32] also suggested microvascular invasion was not associated with posttransplant recurrence. It is important to clarify the degree of microvascular and macrovascular invasion because some investigators included the vessel wall and some investigators included the muscular wall, whereas others only defined microvascular invasion as microscopic tumour invasion identified in the portal or hepatic vein of the surrounding liver tissue. [32], [33] Roayaie et al’s [34] investigation confirmed that invasion of a vessel with a muscular wall predicted HCC recurrence after liver resection.

There are some limitations in the present study. More female donors were observed in the LDLT group. Although previous investigations suggested female-to- male gender match contributed to poor long-term survival, there was little information regarding the influence of donor gender on posttransplant recurrence. [35], [36] Additionally, the largest TTV in the current study was 382 cm^3^. Whether patients with a percentage of lymphocytes no less than 30% and a TTV greater than 382 cm^3^ could achieve good long-term survival was not further studied in the current study.

In conclusion, our study identified TTV and preoperative percentage of lymphocytes as two independent risk factors for patients with HCC undergoing liver transplantation. A prognostic score model based on the two risk factors was proposed in the present study. Our model expanded the Milan criteria without increasing the risk of postoperative recurrence.
